# Newer Features of Adequate Dietaries

**Published:** 1917-07

**Authors:** 


### NEWER FEATURES OF ADEQUATE DIETARIES

The lecture of McCollum of the University of Wisconsin on the supplementary dietary relationships among certain of our naturally occurring foodstuffs, which was given before the Harvey Society of New York and recently published in *The Journal*,[1] illustrates the remarkable innovations which recent years have brought about in the study of the science of nutrition. It reviews the experiences in feeding experiments which have wrought these changes, and indicates why the supposedly ideal plans of a decade or more ago to nourish animals on diets composed of purified protein, carbohydrates, fats and inorganic salts with cellulose as "roughage"—a ration theoretically correct from the standpoint of that time—were doomed to failure.

Ever since Hopkins [2] reported his observation that the addition of small amounts of milk to food mixtures consisting otherwise of purified protein, fats, carbohydrates and salt exerted a favorable influence on nutrition which was out of all proportion to the caloric value of the added milk, it has become more and more probable that there are certain as yet unrecognized substances, present in many of our natural foods, which are indispensable, in an otherwise adequate diet calculated to promote perfect nutrition. The story of how these so-called vitamins have come into prominence as something to be kept in mind and provided for in the construction of the dietary has been told from time to time in these columns [3] as new contributions have been added to our store of facts.

At the present moment the foremost American workers in the study of nutrition are inclined to postulate the need

1 McCollum, E. V.: The Supplementary Dietary Relationships among Our National Foodstuffs, The Journal A. M. A., May 12, 1917, p. 1379.

2 Hopkins, F. G.: Jour. Physiol., 1912, 44, 425.

3 Flavors and Vitamins, Current Comment, The Journal A. M. A., Dec. 26, 1914, p. 2296; Modern Bread and Deficiency Diseases, April 22, 1916, p. 1314; What Is a Vitamin? editorial, May 6, 1916, p. 1470; Dietary Toxicity Versus Deficiency, Oct. 7, 1916, p. 1094.

of at least two types of vitamins, one of which is soluble in fats and is present in such natural products as milk, beef fat, cod liver oil and egg yolk, but not in any notable abundance in the ordinary vegetable oils or the common cereals. The other, water-soluble vitamin, is more widely distributed where active cell structures are found, and is conspicuous in milk and eggs.

The enthusiasm for these new ideas has tended, for the moment, to divert attention from other equally important and perhaps equally novel aspects of the subject. Since various factors essential to the dietary are now clearly recognized, it is quite possible to compound a ration in which one or more of them is lacking. Nutritive disaster then ensues sooner or later. It remains to be seen, says McCollum, whether or not the animals which have been brought experimentally into a condition of nutritive disaster by a series of diets, each of which was faulty in respect to only a single dietary factor, as inorganic salts or protein, or the unknown A or B, will reveal to the histologist and pathologist lesions analogous to those seen in the states of human malnutrition presumably of dietary origin.

In the case of maize, for example, more than a single dietary factor is at fault in determining the failure of young animals to grow on this seed. When suitable inorganic salts, additional protein of an adequate sort, such as the casein of milk, and butter fat with its fat-soluble vitamin are added, successful nutrition becomes assured. All of these additions, representing the remedy of three distinct defects of maize alone, are needed. For wheat a similar story has been evolved, except that the special deficiencies of this seed are somewhat different from those of maize.

By an appropriate selection and combination of natural foods based on a physiologic analysis in the manner briefly indicated, it has at length begun to be possible to accomplish in a somewhat scientific way the construction of dietaries in which two or more products, each inadequate by itself, may supplement each other so as to make an adequate mixture. Many of our common food concoctions are in reality exemplifications of such combinations. But empiricism is by no means a safe guide. It is gratifying to realize that a mode of attack on the problems of suitable combinations is in sight. Meanwhile we must learn to avoid too great dogmatism, the dangers of which have already been indicated in another connection in relation to questions of diet.[4] As McCollum points out:

"The consumption of a varied diet makes in some degree for safety, but will by no means insure safety. * * * The inadequate diets of man are probably never highly satisfactory with respect to all factors but one. Rather they are in some degree unsatisfactory with respect to two or three dietary factors simultaneously, and the probability that they will be so increases greatly as the food supply tends to be limited to the seeds of plants, and such manufactured products as are derived from the endosperm of seeds. The time will doubtless come within a few years when specific advice can safely be given as to the best way to plan a variety of human dietaries; but a consciousness of the paucity of our knowledge concerning the peculiar supplementary relationships among our natural foodstuffs forbids more than a general statement as to the safest policy now."—*Journal American Medical Association.*

